# BRAF vs RAS oncogenes: are mutations of the same pathway equal? differential signalling and therapeutic implications

**DOI:** 10.18632/oncotarget.2555

**Published:** 2014-10-21

**Authors:** Eftychia Oikonomou, Evangelos Koustas, Maria Goulielmaki, Alexander Pintzas

**Affiliations:** ^1^ Laboratory of Signal Mediated Gene Expression, Institute of Biology, Medicinal Chemistry and Biotechnology, National Hellenic Research Foundation, Athens, 11635, Greece

**Keywords:** BRAF vs KRAS, Cancer, differential signalling, targeted therapeutics, rational drug combinations to overcome resistance

## Abstract

As the increased knowledge of tumour heterogeneity and genetic alterations progresses, it exemplifies the need for further personalized medicine in modern cancer management. Here, the similarities but also the differential effects of RAS and BRAF oncogenic signalling are examined and further implications in personalized cancer diagnosis and therapy are discussed. Redundant mechanisms mediated by the two oncogenes as well as differential regulation of signalling pathways and gene expression by RAS as compared to BRAF are addressed. The implications of RAS vs BRAF differential functions, in relevant tumour types including colorectal cancer, melanoma, lung cancer are discussed. Current therapeutic findings and future viewpoints concerning the exploitation of RAS-BRAF-pathway alterations for the development of novel therapeutics and efficient rational combinations, as well as companion tests for relevant markers of response will be evaluated. The concept that drug-resistant cells may also display drug dependency, such that altered dosing may prevent the emergence of lethal drug resistance posed a major therapy hindrance.

## INTRODUCTION

### The MAPK signalling pathway

**Figure 1:**
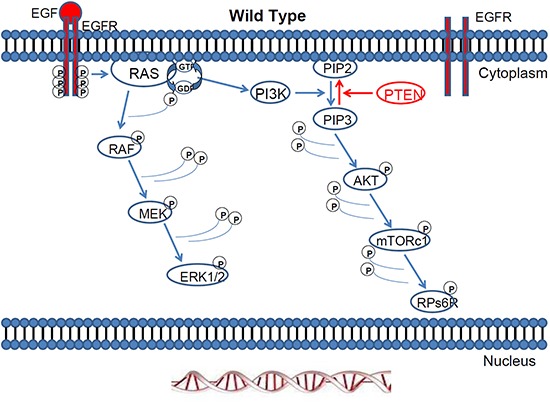
The Ras/Raf/MEK/ERK pathway and the Ras/PI3K/PTEN/mTOR pathway are activated by external factors such as growth factors and mitogens Once RAS is turned on, it recruits and activates proteins necessary for the propagation of growth factor and other receptor signals, such as RAF and PI3K.

Mitogen-activated protein kinase (MAPK) pathways modulate the extracellular signals to control growth, proliferation, differentiation, migration, and apoptosis. One of the most studied MAPK pathways is the extracellular signal-regulated kinase (ERK) pathway. ERK is a subgroup of MAPKs that is activated by external factors such as growth factors and mitogens. Ligand-mediated activation of receptor tyrosine kinases like Epidermal growth factor receptor (EGFR) initiate the cascade of ERK signalling that flows through RAS GTPase, which acts as a molecular on/off switch [1]. Once RAS is turned on, it recruits and activates proteins necessary for the propagation of growth factor and other receptor signals, such as RAF and phosphatidylinositol 3-kinase (PI3K). RAF activation is achieved through a complex process that requires lipid and protein binding, conformational changes, and regulatory phosphorylation and dephosphorylation events. There are three RAF proteins in mammals, ARAF, BRAF, and CRAF, and they can all activate MAP kinase kinase (MEK) just upstream of ERK but they clearly perform distinct functions *in vivo* as shown by the phenotypic differences between ARAF, BRAF and CRAF null mice [2]. When the EGFR pathway is activated, small G-protein RAS acts through protein kinase RAF and activates the MAPK cascade [3, 4] Figure 1.

## KRAS VS BRAF ONCOGENIC SIGNALLING

### KRAS mutation

In most tumour types exhibiting mutation of a RAS gene family member (HRAS, KRAS, or NRAS), the mutational activation of one member predominates. In solid tumors, including colorectal, lung and pancreatic cancer, KRAS is mutated much more frequently than NRAS; the reverse is true in some hematologic cancers such as acute lymphoblastic and chronic myelomonocytic leukemias, and Hodgkin lymphoma [5], (Table 3). Approximately 90% of the activating mutations are found in codons 12 (wild-type GGT) and 13 (wild-type GGC) of exon 1 and ~5% in codon 61 (wild-type CAA) located in exon 2 (8–10). The most frequently observed types of mutations are G>A transitions (G12D: GGT → GAT) and G>T transversions (G12V: GGT → GTT) in codon 12 and G>A transversion (G13D: GGC → GAC) in codon 13 [198]. In addition, although KRASG12D seems to be more frequent compared with KRASG12V in colon cancer, G12V has been associated with more aggressive colorectal carcinomas and greater mortality than other codon 12 or 13 mutations. KRAS activating mutations are widely recognised as predictors of resistance to the treatment with anti-EGFR monoclonal antibodies (moAbs) in metastatic colorectal cancer (mCRC) patients [6, 7]. Additional KRAS-activating mutations, involving codons 61 and 146 on exon 3 and 4 respectively were identified at amino acid residues Q61 and A146 [8] and occur with frequencies ranging from 1 to 4% in CRCs. These relatively rare mutations, as well as codons 12 and 13 mutations, are responsible for the oncogenic constitutive activation of RAS/RAF/MAPKs pathway [9]. Several studies have examined the predictive value of KRAS mutation in codon 61 and/or 146 in metastatic colorectal cancer (CRC) treated with anti-EGFR therapy. Lately the same value was establisehed for NRAS condon 61 mutation. Both KRAS and NRAS mutations have been observed to be associated with primary resistance to EGFR blockade when they occur in primary CRCs [10, 11].

Although some data suggest potentially distinct biological consequences for mutation of the related RAS family members, studies demonstrating a clear clinical distinction between *NRAS* and *KRAS* are lacking. In general, the mutual exclusivity of mutations of *NRAS* and *KRAS* in varied tumor types suggests that they provide similar or identical oncogenic signals. While NRAS and KRAS may be capable of equal signaling through the RAF/MAPK pathway, there is growing evidence suggesting that NRAS mutation also provides a distinct, prosurvival signal that mutational activation of KRAS does not [12, 13].

What is interesting about KRAS mutations is that in pancreatic cancer the most common mutation is one amino-acid substitution in position 12 of the KRAS protein, leading to a glycine (G) to aspartic acid (D) substitution, although other variants, such as G to V are also common [14]. The highest incidence of KRAS mutations are found in adenocarcinomas of the pancreas (90%), with activating point mutations in codon 12 of KRAS to be the most common oncogene alterations [15]. From early on has been speculated that for the induction of pancreatic tumours a single activated RAS gene is a critical if not sufficient event [16].

Many studies have indicated that KRAS mutations are found earlier in CRC. Mutations in KRAS and BRAF are mutually exclusive, but KRAS and PIK3CA mutations may coexist within the same tumor [17, 27]. Poor prognosis and significant association with Dukes' stage D suggest that tumours with KRAS and PIK3CA mutations are more likely to develop into liver metastasis [18]. The molecular significance and therapeutic implications of co-occurring mutations are unclear, but the fact that both genes are acting on the same pathway, suggests a possible synergistic effect on the signalling pathways controlled by these genes during CRC development. Additional mutations within the same pathway may enhance the oncogenic transformation by strengthening PI3K pathway signaling caused by oncogenic RAS, thus activating other pathways.

### BRAF mutation

Among the BRAF mutations observed in melanoma, over 90% are in codon 600, and among these, over 90% are a single nucleotide mutation resulting in substitution of glutamic acid for valine (BRAFV600E: nucleotide 1799 T>A; codon GTG>GAG). The second most common mutation is BRAFV600K substituting lysine for valine, that represents 5–6% (GTG>AAG), followed by BRAFV600R (GTG>AGG), an infrequent two-nucleotide variation of the predominant mutation, BRAFV600′E2′ (GTG>GAA), and BRAF V600D (GTG>GAT) [Catalogue of Somatic Mutations in Cancer (COSMIC) [http://www.sanger.ac.uk/cosmic]. The prevalence of BRAFV600K has been reported as higher in some populations [19].

### KRAS and BRAF deregulated MAPK signalling

**Figure 2:**
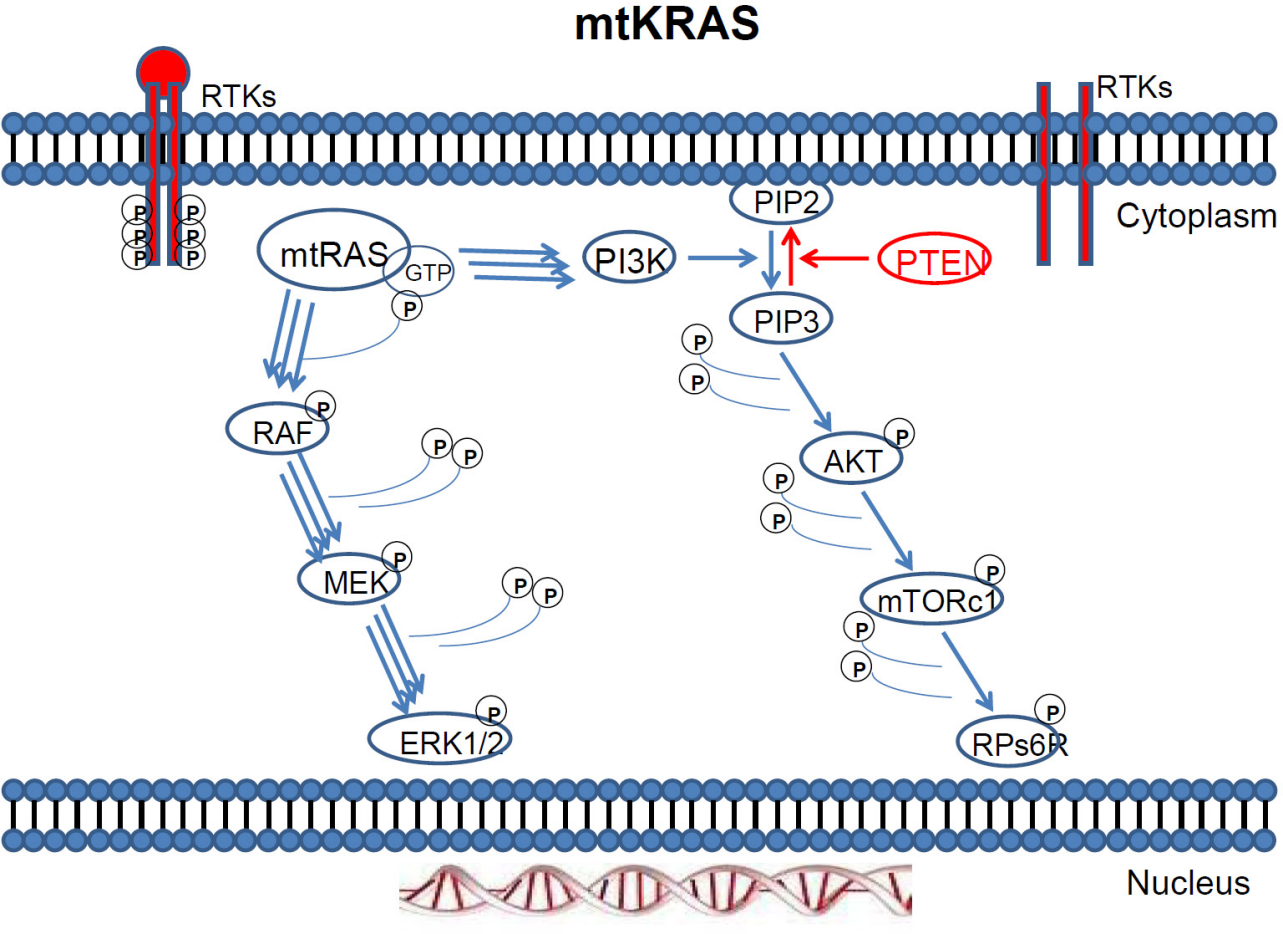
Mutant KRAS two way activation of the MAPK and PI3K pathway

**Figure 3:**
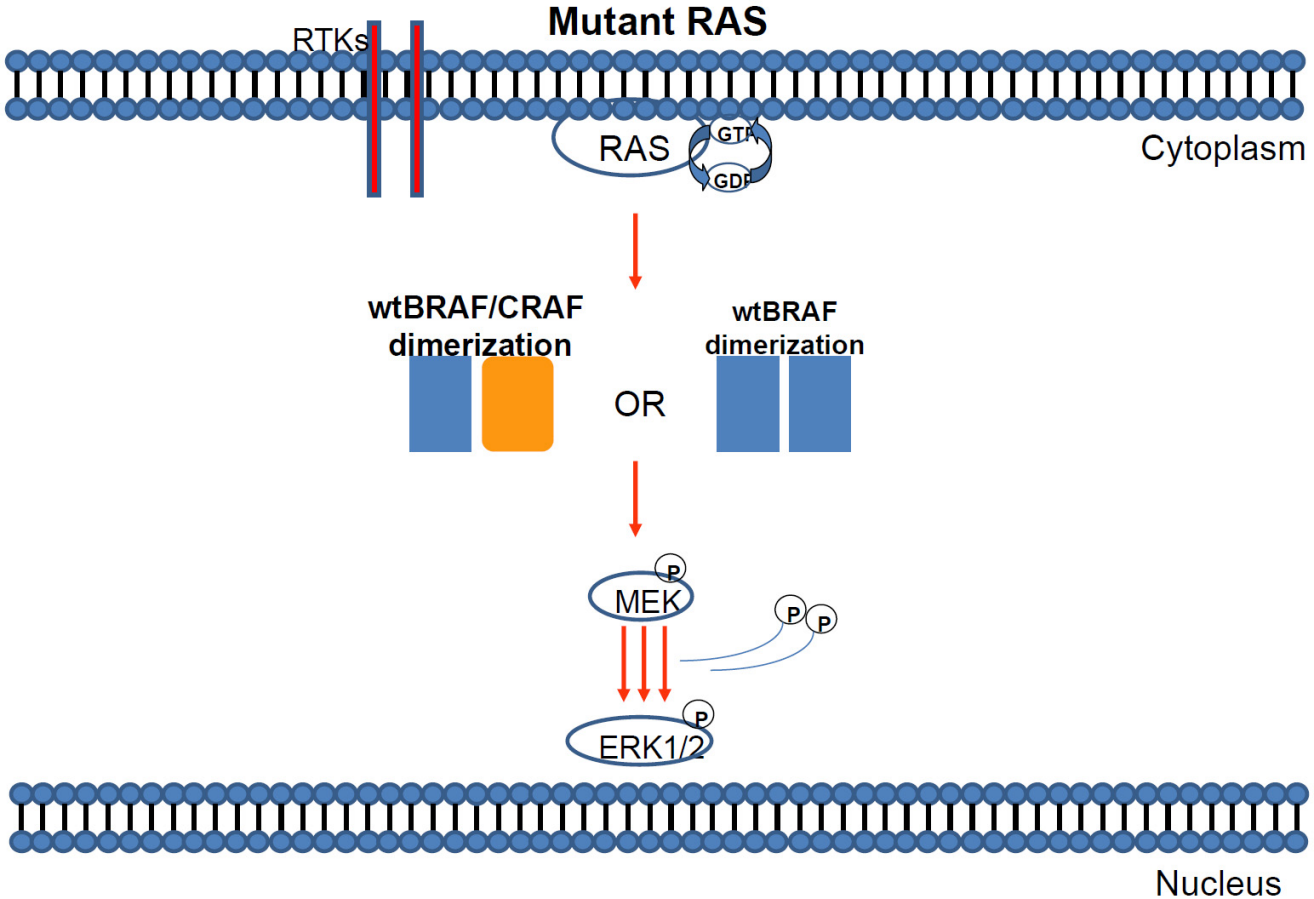
Mutant KRAS can induce either BRAF/BRAF or BRAF-CRAF dimerization of wild type proteins

**KRAS** gene mutations have been reported in approximately 15–30% of human solid tumours, where the MAP kinase (MAPK) pathway is found hyperactivated [20, 180] (Figures 2–3). This mutation is the most common abnormality of dominant oncogenes in human tumours [21] and is a common event in the development and progression of adenocarcinomas of the pancreas (90%), colon (50%), thyroid (50%), bladder (50%), and lung (30%). The RAS family of genes is of particular interest in Head and Neck Squamous Cell Carcinoma (HNSCC) because a mechanism for mutation (activation) of KRAS by tobacco carcinogens has been suggested [22, 23]. Furthermore, RAS mutations have been observed in other tobacco-related cancers, namely, pancreatic carcinoma and non-small cell lung carcinoma [22]. The frequency of *KRAS* mutations in colorectal cancer (CRC) ranges from 24% to 50% depending on the study and the sample source [24, 25].

**Figure 4:**
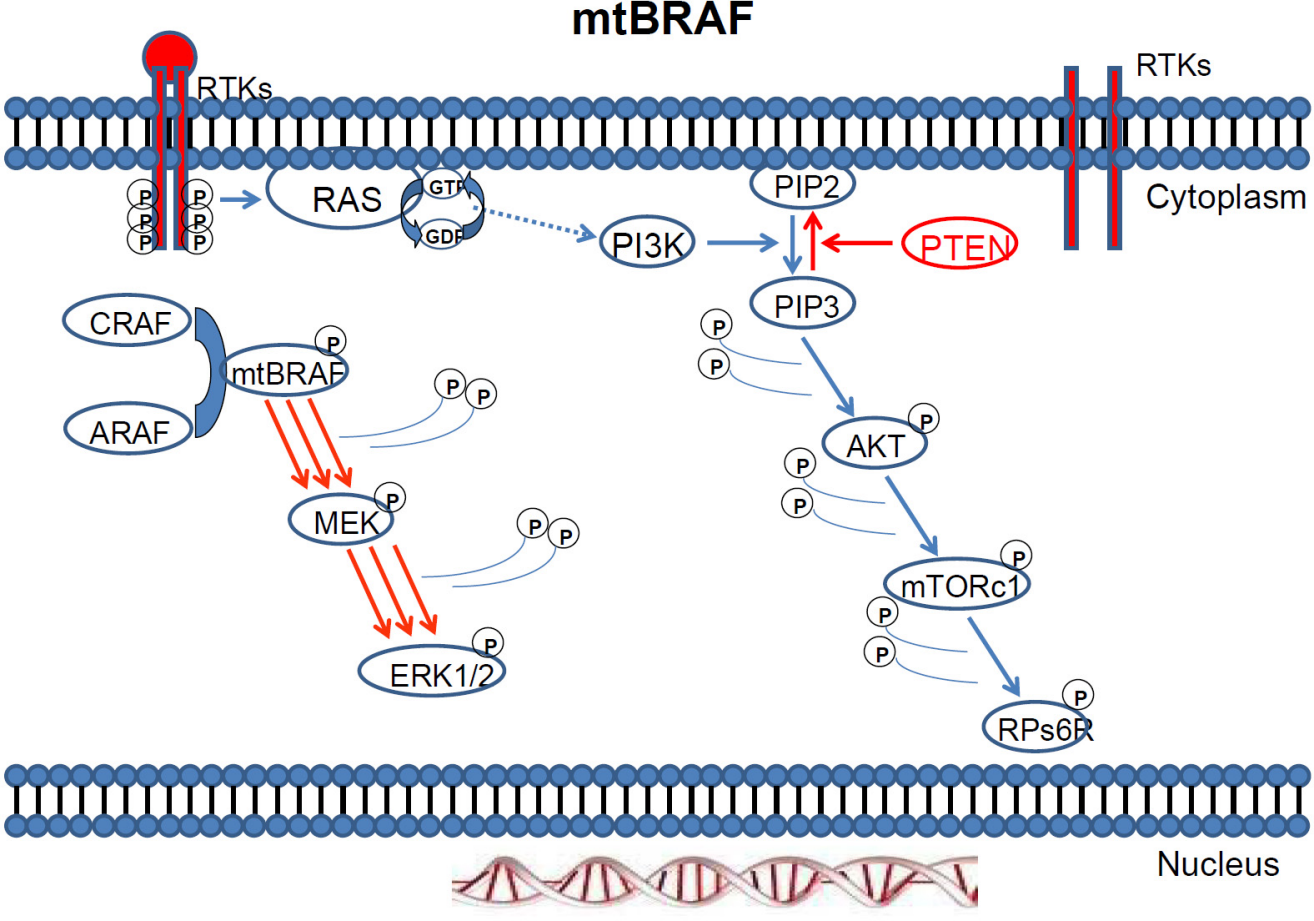
MAPK pathway activation in response to BRAF mutation

**Figure 5:**
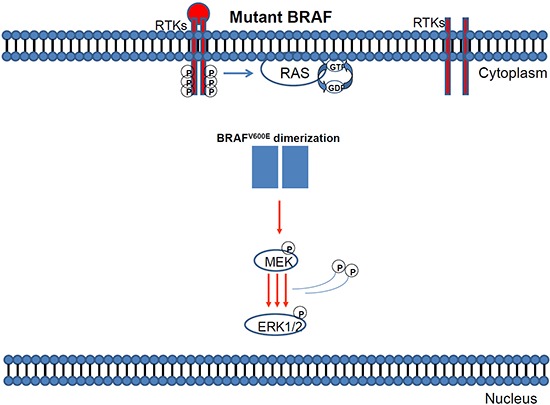
Mutant BRAF activation via protein dimerisation

Mutations in ***BRAF***, the downstream effector of KRAS are reported in up to 70% of melanoma cancer cell lines [27] and they are highly prevalent in most common cancers with poor prognosis such as malignant melanoma [27, 28, 180] (Figures 4–5). Mutations in *BRAF* have been reported in up to 60% of melanoma cases, between 40 to 70% of thyroid carcinomas, and up to 18% of CRCs [29].

Several studies have reported a range of frequencies regarding *BRAF* mutations in CRC (4%–18%) [24, 27, 30, 31]. BRAF mutations have long been connected with microsatellite instability (MSI) in sporadic colorectal tumours, because mismatch repair-deficient tumours have a very high incidence of BRAF mutations. mtBRAF overexpression in a rat cell line of thyroid origin (PCCL3) and human colon adenocarcinoma cells, induced chromosomal instability (CIN) and MSI respectively. Less common is the presence of BRAF mutations in micro-satellite stable (MSS) tumours, indicating poor prognosis in the latter [31–34].

In cancer models: The relative significance of BRAF and RAS mutations in human tumours have been validated in many relevant cancer models. Mutations in KRASG12V and subsequent overexpression in human colon adenocarcinoma cells results in high ERK activation. Since ERK can phosphorylate and regulate functions of numerous cellular components, its constitutive activation leads to cell transformation and activation of the senescence programme at the same time. This controversy suggests a diminished KRAS transforming capability as compared to mutations occurring to another downstream effector component BRAF [19]. In contrast, the same mutation in HRAS isoform, is far more potent in transforming human colon intermediate adenoma cells with induction of Epithelial-Mesenchymal Transition (EMT) [26]. By itself, BRAFV600E mutation shows a 138-fold transforming and oncogenic activity over BRAFWT [27] and is a more potent oncogene than KRASG12V [34].

Both KRAS and BRAF mutations fall into the “driver mutation” classification, since it appears to be one of the first events in the malignant transformation to cancer [35]. However BRAF has also been characterised as a “gatekeeper mutation” since the mutation that occurs in the ATP binding site of the BRAF protein kinases mediate resistance to small molecule inhibitors. Gatekeeper mutations have also been detected in EGFR and PIK3CA proteins [36, 37]. Terms like “oncogene addiction” have always characterized activating mutations in BRAF and PIK3CA likewise inactivation of PTEN gene. The cells become addicted to the consequences of that mutation and grow under conditions where a normal cell would terminate. Many malignant melanoma cells become addicted to mutant BRAF for proliferation. Likewise either mutation of PIK3CA or silencing of PTEN and subsequent activation of AKT is a frequent form of oncogene addiction in many tumour types [38, 39].

### The RAS/RAF/MEK/MAPK signalling pathway

The RAS/RAF/MEK/MAPK signalling pathway downstream of EGFR plays a significant role in tumourigenesis.

In Cancer models: The identification of mutationally activated KRAS and BRAF alleles in several tumour models supports the importance of this signalling pathway in cancer progression [27, 33]. Several reports have shown that MAPK activation, owing to oncogenic RAS and BRAF mutations, is likely to be involved in promoting cellular invasiveness in different tumour models [40–43]. In sporadic CRC lesions, KRAS mutations are inversely associated to the oncogenic BRAFV600E mutation [33, 44–46], suggesting that each mutation can induce similar cellular effects and signal through the same pathway. Presumably, in these tumour stages, BRAF mutations do not occur concominantly with KRAS mutations because their combined signalling is incompatible with proliferation, as an excess ERK signalling could lead to cell cycle arrest, differentiation, senescence or even cell death, as shown in cancer models [47-50].

## FUNCTIONAL ROLES OF RAS AND BRAF ONCOGENES DURING CANCER PROGRESSION AND METASTASIS;STUDIES IN CANCER MODELS

The significance of RAS oncogenes in cancer progression has been shown in many studies involving tumour models. HRASV12 can transform immortalized cells, accompanied by activation of MEK and PI3K pathways [51], whereas overexpression of the same oncogene in normal cells causes cell cycle arrest and senescence [52], which in other systems is associated with a negative feedback signaling network [34, 53]. Moreover, regulated expression of RAS in cell and animal models results in the appearance of transforming and tumourigenic properties, which are dependent on the tumour model context [54–57]. In pancreatic cancer, KRAS promotes growth transformation and invasion of immortalized human pancreatic cells by Raf and PI3K signaling [58], while the same mutation in mice causes early onset lung cancer [59]. Pathway activation between EGFR family- and KRAS-dependent tumourigenesis are comprehensively reviewed in two review papers [60, 61]. In CRC, oncogenic KRAS is required to maintain changes in cytoskeletal organization, adhesion, and motility of colon cancer cells [62]; however, differential effects of oncogenic KRAS and N-RAS have been reported on proliferation, differentiation and tumor progression in the colon [63]. Oncogenic RAS co-operates with other oncoproteins, as in the case of requirement for interaction of PI3-kinase p110α with RAS in lung tumour maintenance [64], or the study where single copies of mutant KRAS and mutant PIK3CA cooperate in immortalized human epithelial cells to induce tumour formation [65].

The differential biological effect of RAS versus BRAF oncogenes has been the topic of several studies. In CRC, they differentially induce tumour cell properties [34], global gene expression [66] and they differentially regulate hypoxia-inducible factor-1alpha and -2alpha [67]. On the other hand, both KRAS and BRAF oncogenes inhibit colon epithelial polarity establishment through up-regulation of c-myc [68], and both oncogenes promote expression of dual-specificity phosphatase 4 (DUSP4/MKP2) and of the stem cell marker CD133 [69, 70].

On the other hand, the role of BRAF oncogene in tumourigenesis has been extensively described in melanocytes. Early studies have indicated BRAFV600E-associated senescence-like cell cycle arrest of human naevi [71–73], which can later develop melanomas in mice [74]. More recently, a mouse model of intestinal carcinogenesis is driven by BRAFV600E oncogene, which initiates an alternative pathway to colorectal cancer (CRC) and progresses through a hyperplasia/adenoma/carcinoma sequence [75]; or after BRAFV600E having induced gastrointestinal crypt senescence [76].

In summary, KRAS and BRAF have key roles as driver oncogenes in tumourigenesis in many different types of tumours and depending on the tumour type they can differentially regulate key pathways and genes to provide differential biological effects. Last but not least, they can co-operate with other genes for more profound tumour phenotypes.

## DIFFERENTIAL IMPACT OF KRAS VS BRAF ONCOGENES IN DISEASE DIAGNOSIS, PROGNOSIS AND OUTCOME

### BRAF oncogene is a marker of poor prognosis in Sporadic Colorectal Cancer (CRC)

Sporadic CRC development is a multi-step process involving MSI, mutations in APC, SMAD4, KRAS, TP53 and b-catenin. KRAS and BRAF mutation frequency is similar in stage II and III CRC.

**KRAS:** Oncogenic mutations affecting the RAS GTPase molecular on/off switch, have been closely associated with the development of sporadic CRC, in about 35%-45% of the cases and codon 12 and 13 are two hotspots, which account for about 95% of all mutation types, with approximately 80% occurring in codon 12 and 15% in codon 13 [77, 78]. **Location:** KRAS-mutated carcinomas are distributed in a bimodal pattern along the proximal-distal axis of the colorectum, are frequently associated with a residual polyp and show molecular features distinct from other colorectal carcinomas, in particular from tumours with neither BRAF nor KRAS mutation. Compared with male subjects, female subjects are more likely to have KRAS-mutated carcinoma in the transverse colon and descending colon. No difference in overall survival (OS) was observed in patients according to their tumour KRAS mutation status in total [79]. **Prognostic value:** It is generally accepted that in stage II-III CRC, the KRAS mutation status does not have a major prognostic value [80]. Nevertheless, differences in *KRAS* mutations at codons 12 and 13 may result in different biological, biochemical and functional consequences that could influence the prognosis of CRC. KRAS codon 12 mutations (in particular, c.35G>T), but not codon 13 mutations, are associated with inferior survival in BRAF wild-type CRC. This data highlight the importance of accurate molecular characterization in CRC [81]. In another study, patients with p.G13D-mutated tumours have a worse OS in patients with advanced and recurrent CRC than tumours with either KRAS wt or bearing another KRAS mutations [82]. Likewise, KRAS splice variants: KRAS4A and KRAS4B and their relationships with various clinico-pathological characteristics in CRC have been investigated. Interestingly, KRAS4A overexpression was associated with a better OS, while overexpression of the KRAS4B variant was significantly associated with larger tumour size [83], Table 1.

**Table 1:** Prognostic value of RAS and BRAF mutations

Cancer type	Mutated gene	Model/Study level	Results	Reference
**Melanoma**	BRAFV600E	Mouse/preclinical	Large pigmented perianal lesions,melanocytic lesions in the eyelids, large pig mented perianal epithelioid blue nevi	*Dhomen et al., Cancer Cell, 2009 [202]*
**Colorectal stage II/III**	KRASBRAF	Human/clinical phase III	Not a major prognostic role for overall survivalPrognostic role for overall survival in MS-L/S tumors	*Roth et al., J Clin Oncol, 2010 [203]*
**mt Colorectal**	BRAF	Human/clinical	Predictive biomarker for poor prognosis in mCRC patients undergoing anti-EGFR MoAbs therapy	*Yuan et al., PLOS One, 2013[204]*
**mt Colorectal**	BRAFV600E	Human/clinical	Risk of mortality is increased by 1.7 times in patients bearing this mutation.	*Safaee Arkedani et al., Plos One 2012 [96]*
**mt Colorectal**	KRAS G13D	Human/clinical	Poor prognosis for overall survival compared to other KRAS mutations or KRAS wild type tumors	*De Roock, JAMA 2010[82]*
**mt Melanoma**	BRAFV600ENRAS	Human/clinical	BRAF mutation is a weak prognostic factor but a strong predictive factorBetter prognostic value compared to the BRAF mutation	*Ekedahl et al., Br J Dermatol., 2013 [205]*
**Melanoma Stage IV**	NRASBRAF	Human/clinical	The presence of an NRAS mutation correlates with shorter survival from the diagnosis of stage IV melanoma. The presence of either a BRAF or NRAS mutation is associated with an increased risk of CNS involvement at initial stage IV diagnosis.	*Jakob et al, Cancer, 2012 [206]*
**Lung adenocarcinoma stage I**	KRAS	Human/clinical	K-ras mutations were an independent poor prognostic factor. Overall survival was significantly shorter in patients with KRAS mutations	*Ohba et al., Surg Today, 2013 [207]*
**Papillary Thyroid Carcinoma**	BRAFV600E	Human	A high percentage of BRAF(V600E) alleles predicts a poorer disease outcome.	*Guerra et al., J Clin Endocrinol Metab., 2012 [208]*

BRAF: Mutations affecting the RAS effector, BRAF oncogene, have been implicated only in 10% of the cases of CRC and never in association with KRAS mutations [33, 44, 84, 85]. So far, over 50 distinct mutations have been identified in the *BRAF* gene. Of all *BRAF* activating mutations, a point mutation results in a valine to glutamic acid substitution a (T-A), also known as *BRAFV600E*, is the most common change. In fact, this single mutation dramatically increases BRAF activity and accounts for more than 80% of all reported *BRAF* mutations in tumours and implicates the constitutive activation of BRAF [89]. **Location:** BRAF mutated tumours are more likely to develop on the right of the colon, and appear as poorly differentiated adenocarcinomas or mucinous carcinomas, as well as with peritoneal metastasis, compared with left side CRC [78–90]. Likewise, a higher frequency of MSI especially of the MSI-High phenotype, which is a poor prognostic factor for CRC, has been reported to be more prevalent in right side compared with left side in the BRAF mutant tumours [91–93]. BRAF appears to be a strong prognostic factor for OS, particularly in MS-L/S stage II patients [80]. **Prognostic value:** BRAF mutations emerged as an independent prognostic factor for both progression free survival (PFS) and OS, comprising one of the most powerful prognostic factors for advanced and recurrent CRC [93]. BRAF mutation is likely to be a convenient marker for the identification of a subset of CRCs with distinctive clinical, pathological and molecular features which may originate in hyperplastic polyps and serrated adenomas, while increases the risk of mortality in CRC patients by more than two-fold [95]. In addition, it was revealed that *BRAFV600E* mutation also increases the risk of mortality in melanoma patients by 1.7 times, while its effect on papillary thyroid carcinoma still requires further investigation [96], Table 1.

In summary, KRAS is a more frequent mutation than BRAF in CRC mostly distributed along the proximal-distal axis of the colorectum, has little prognostic value as compared to BRAF in overall patient survival as compared to BRAF, the latter being a mutation more likely to develop on the right of the colon. BRAF is highly associated to metastasis, MSI events and appears to be a strong prognostic factor for overall patient survival.

### BRAF and not KRAS oncogenic mutations are associated with MSI in CRC

Microsatellite instability is defined as small deletions or expansions within short tandem repeats in tumour DNA, which resulted from the inactivation of the DNA mismatch repair (MMR) system and characterised by the absence of protein expression encoded by the corresponding MMR genes (hMLH1, hMSH2, hMSH6 or PMS2) [97–100]. Substantial progress has been made to identify causes of chromosomal instability in colorectal cells and to determine the effects of the different forms of genomic instability on the biological and clinical behaviour of colon tumours.

BRAF: In CRC tumours, BRAF mutations seem to occur more frequently in cases with MSI characterised by deficient DNA mismatch repair (dMMR) [32]. Approximately only 5% of microsatellite stable (MSS) CRC cases also show mutations within BRAF gene [43, 44, 85, 100]. BRAF has been associated with dMMR CRCs, with approximately 40% of MSI-high tumours having a BRAF mutation compared to nearly 5% of MSS tumours [91, 92]. Moreover, several studies suggest that the *BRAFV600E* mutation occurs much more frequently in MSI-High tumours in comparison with MSS tumours [46, 103] and can be the cause of conversion of CIN to MSI in colorectal tumour cells [34]. BRAF appears to be strong prognostic factor for OS, particularly in Microsatellite instability low (MSI-L) /stage II patients [54], Table 1.

KRAS: Initially, KRAS mutations have been observed in colorectal tumours independently of their MSI status [104–106]. However, a more recent study supports that even though KRAS tumour mutation status has no major prognostic value for relapse-free and OS in patients with stage II and III CRC treated with adjuvant chemotherapy, it may actually have slightly significant prognostic value when observed in the multivariate analysis of the MSI-L/ and MSS subpopulation [80]. At this study the different effect of a KRAS mutation in MS-L/MSS and MSI-High tumours was consistent with the concept that these are different forms of CRC [104, 107]. In a large study of the prognostic and predictive value for patients deficient in MMR, KRAS and BRAF provided additional useful risk stratification in rectal and possibly CRC. Patients proficient in MMR and wild-type for KRAS have an intermediate prognosis whereas those with KRAS mutations have the highest recurrence rate [108], Table 1.

In summary, BRAF mutations appear to occur more frequently in CRC tumours with MSI-high characterised by dMMR, posing as strong OS prognostic factor, but also presence of KRAS mutations in patients proficient in MMR may suggest highest recurrence rate.

### Tracing origin of serrated adenomas with BRAF and KRAS mutations

Emerging evidence supports the existence of an alternative pathway of CRC development through the serrated polyp. Molecular studies have indicated that serrated polyps are likely to be clonal neoplasms, because mutations in KRAS and p53, MSI and chromosome 1p loss have been found in variable and low incidence as compared to sporadic tubular adenomas. However, mutations of adenomatous polyposis coli are uncommon [109, 110]. Activation of the RAF/RAS signal transduction cascade by RAS mutations in serrated neoplasia is mainly observed in hyperplastic (serrated) polyps, and their occurrence in traditional type serrated adenoma is limited, indicating that activation of the RAS/RAF/MEK/ERK/MAP kinase pathway constitutes a highly significant event in the pathogenesis of this group of lesions especially in the early steps in the initiation of the serrated neoplasia pathway [94, 101, 102]. Interestingly BRAF mutations occurred only in polyps with the serrated architecture and show a similar frequency with that in MSI CRC, supporting that BRAF activation is pivotal in the serrated pathway of CRC [113–116].

### Oncogenic RAS and BRAF signatures in CRC

Microarray analysis allows classification of cancers to predict the prognosis or therapeutic responsiveness of cancer patients [117–119, 120, 121]. In CRCs, microarray analysis was used to divide samples into two groups based on the existence of MSI, which is an important marker in CRC [104]. Recently, BRAF mutant melanoma samples were distinguished from BRAF wild-type samples [123], suggesting that gene expression profiling according to BRAF status might be useful for the identification of molecular markers involved in RAS-RAS-MEK-ERK-MAPK signalling. Identifying the model of activated EGFR-signalling pathways in patients by studying gene expression pattern can lead to the development of targeted therapies or even may predict the response of individual patients to EGFR pathway inhibitors.

In cancer models: BRAF and RAS oncogenes are considered to induce cancer properties by regulating similar signalling pathways. Microarray data derived from these studies can validate and further interprete data derived by analysis of clinical samples. Comparison between BRAFV600E signature analyzed [124] with another study where a RASV12 signature was demonstrated [125], shows that their gene expression signatures can pinpoint interesting mechanistic differences between these two frequently mutated oncogenes in CRC. Regarding apoptosis and nucleotide excision repair, oncogenic BRAF upregulates caspase 6 and downregulates XPC and ERCC1, which is not the case for oncogenic KRAS [124, 125].

### KRAS and BRAF response signatures

In a different study, independent gene expression profile analysis of diverse oncogenic mutations in BRAF or KRAS uncovered signatures of activated EGFR pathway signalling [126]. While the combined oncogenic pathway signature correctly predicted the presence of a mutation in KRAS or BRAF known mutation carriers, interestingly, more than two fold of the tumours that had no oncogenic mutations in BRAF and KRAS were also classified as oncogenic based on their gene expression signatures indicating that many KRAS and BRAF wild type patients share the same phenotype of an activated EGFR pathway as the patients with at least one activating mutation. From a biologic perspective, this finding supports the notion that the poor outcome of tumours with BRAF mutation is shared with some non-BRAF-mutated tumours, suggesting that they have common biology driving poor survival after relapse [127]. Moreover, as shown in the original publication [128], KRAS mutation status is an indicator of response with wild type patients having a better outcome than patients with activating mutations [126].

## RAS VS BRAF ONCOGENES AND TARGETED THERAPIES

### Prognostic Value of KRAS/BRAF to therapies in Sporadic CRC. KRAS and BRAF as Pharmacological Targets

RAS and several of its downstream effectors, including BRAF, have been shown to be commonly mutated in a broad range of human cancers and biological studies have confirmed that RAS pathway activation promotes tumour initiation, progression and metastatic spread in many contexts. Current efforts concentrate on developing inhibitors of RAS and its key downstream effectors, BRAF, MEK, ERK [127, 130] as well as on the need for mutational testing of RAS/BRAF before proceeding to the clinical setting, Table 1.

**RAS:** RAS has been the obvious target for anticancer therapy since is frequently mutated in human cancer. Because of the high affinity between RAS–GTP interaction, instead of a small molecule inhibitor, the non-selective farnesyl tranferase inhibitors were developed, aiming to inhibit post-translational events. However these proved to be unsuccessful due to redundant regulation of KRAS by geranylgeranyl transferase [131, 132]. In a recent study, a common mutant form of KRAS, bearing a glycine-to-cysteine substitution at codon 12 was successfully targeted. It is detected in particularly high rates in lung cancer, occurring in 40% of KRAS mutant lung tumours. This is the first piece of work to report the development of small molecules that irreversibly bind to a common oncogenic mutant, KRASG12C and provides structure-based validation of a new allosteric regulatory site on Ras that is targetable in a mutant-specific manner [133].

**BRAF:** Alternative approaches involving inhibitors of the key downstream effectors of RAS were therefore pursued. RAF, MEK, and ERK, all downstream RAS effectors, are cytosolic protein kinases that form a tiered protein kinase cascade downstream of RAS [116]. The limited activity-selectivity of Sorafenib (BAY 43–9006), the first RAF kinase inhibitor, for the RAF kinases [134–137] in tumours with BRAF mutation prompted the development of second-generation RAF inhibitors with greater selectivity for mutant BRAF. Sorafenib is currently undergoing clinical evaluation for a variety of additional cancers, including non-small cell lung cancer [138]. The selective BRAF inhibitor Vemurafenib (PLX4032/R7204) and its analog PLX4720, by far the most advanced in clinical studies, have shown potent antiproliferative effects in several preclinical models specifically in cell lines harboring BRAFV600E mutations [139, 140]. Phase II trials are currently enrolling patients with metastatic papillary thyroid cancer for treatment with vemurafenib, and with locally advanced disease using vemurafenib as a neo adjuvant approach with which to improve surgical resectability. GSK2118436 (Dabrafenib) another ATP competitive BRAF inhibitor, has recently shown a dramatic effect as single agent in patients with metastatic melanoma and other solid tumours [143, 153], and is currently under a phase I/II clinical trial [145]. Remarkably, Vemurafenib and Dabrafenib are currently FDA approved drugs against BRAFV600E positive metastatic melanomas [179].

The XL281 (BMS-908662) has shown modest biological activity in advanced or mCRC with BRAF or KRAS mutations, alone or in combination with cetuximab [146, 147], Table 2.

**Table 2:** BRAF and downsteam MEK as therapeutic targets

Inhibitor/Antibody	Target	Cancer type	Clinical phase	ClinicalTrials.gov Identifier	Outcomes/most common side effects
**Vemurafenib (PLX4032)**	B-RAF, BRAFV600E	Malignant Melanoma	Phase II complete	NCT01248936	No results posted
		Solid tumors, multiple myeloma	Phase II recruiting	NCT01524978	No results posted
		Colorectal cancer, melanoma	Phase I complete	NCT00405587	No results posted
**Sorafenib (BAY 43-9006)**	B-RAF, C-RAF, VEGFR,PDGFRb	Hepatocellular carcinoma	Phase IV recruiting	NCT01203787	No results posted
		Advanced Solid tumors	Phase I complete	NCT00941863	Partial response and progression blockade in higher doses/Blood components' abnormalities
		Renal cell carcinoma	Phase III complete	NCT00478114	
		Non-small cell lung carcinoma	Phase II complete	NCT00064350	1/3 of patients showed an increase in overall survival/Anemia, diarrhea, dyspepsia, nausea, fatigue, leukopenia, thrombocytopenia, anorexia
**Dabrafenib (GSK2118436)**	B-RAF	Metastatic melanoma	Phase II active	NCT01153763	Increase in overall survival from 8-11 months/Anemia, pyrexia, arthralgea, hyperkeratosis, nausea, fatigue, basal and squamous cell carcinoma
		Solid tumors	Phase I complete	NCT01262963	No results posted
		Non small cell lung carcinoma	Phase II recruiting	NCT01336634	No results posted
**LGX818**	B-RAFV600E	Metastatic Melanoma	Phase I recruiting	NCT01436656	No results posted
**RAF265**	B-RAF,VEGFR-2	Advanced or metastatic Melanoma	Phase I active	NCT00304525	No results posted
**XL281**	B-RAF, BRAFV600E, C-RAF	Solid tumors	Phase I complete	NCT00451880	No results posted
**RO5212054 (PLX3603)**	B-RAFV600	Solid tumors	Phase I active	NCT01143753	No results posted
**Trametinib (GSK1120212)**	MEK1/2	Solid tumors, Lymphoma	Phase I complete	NCT00687622	Median progression-free survival 5–7 months/dermatitis acneiform, diarrhoea
		Oral cavity squamous cell carcinoma	Phase II recruiting	NCT01553851	No results posted
**Pimasertib (MSC1936369B)**	MEK1/2	Solid tumors	Phase I active	NCT00982865	No results posted
**Selumetinib (AZD6244)**	MEK1/2	BRAF mutant cancers	Phase II active	NCT00888134	No results posted
**Dabrafenib-+Trametinib**	BRAF+MEK1/2	Melanoma	Phase III active	NCT01584648	High percentage of patients showed either complete or partial response. Progression Free Survival of approximately 9 months/Not serious side effects. Most common anaemia and pyrexia

**Table 3:** Most frequent codon mutations in BRAF and RAS genes and tissue localization (COSMIC-September 2014)

Mutated gene	Most frequent codons mutated	Most frequent mutations	Most frequent tissues affected
**KRAS**	12	G12D, G12V, G12C	Colorectal, pancreas, lung, Haemato/lymphoid
	13	G13D	colorectal, prostate, ovary, endometrium
	61	Q61H	Colorectal, pancreas, lung, haemato/lymphoid, endometrium
**HRAS**	12	G12V	Skin, thyroid, urinary tract, upper
	13	G13R	Skin, thyroid, urinary tract, upper aerodigestive tract, soft tissue
	61	Q61R	Skin, thyroid, urinary tract, salivary gland, aerodigestive tract
**NRAS**	12	G12D	Haematolymphoid, colorectal, skin
	13	G13D	Haematolymphoid, skin
	61	Q61R, Q61K, Q61L	Skin, thyroid, haematolymphoid, colorectal
**BRAF**	600	V600E, V600K	Thyroid, Skin, colorectal

### Other RAF inhibitors

LGX818 was tested in advanced solid tumours with BRAFV600E mutations, alone or in combination with a MEK inhibitor [151]; PLX3603 in advanced solid tumours with BRAF mutations (Hoffmann-La R). RAF265, an oral highly selective inhibitor of RAF, including BRAF and CRAF has been used in advanced solid tumours with BRAFV600E mutations, alone or in combination with the MEK inhibitor, MEK162 (Novartis Pharmaceuticals) [152]; RO5185426 in unresectable or metastatic papillary thyroid cancer harboring a BRAF mutation and resistant to radioactive iodine therapy as a single agent therapy (Hoffmann-LaR) and the GSK2118436 (Dabrafenib) in advanced stage metastatic NSCLC with a BRAFV600E mutation that progressed after platinum chemotherapy as a single-agent therapy (GlaxoSmithKline), as well as in BRAFV600E metastatic melanoma [153, 183] Figure 5/Table 2.

In total, clinical trials using BRAF inhibitors have indicated some encouraging results for patients bearing BRAFV600 mutations, including an increase in progression free and/or overall survival (clinicaltrials.gov; NCT00941863, NCT00064350, NCT0115376, Table 2). Similar results have been demonstrated after treatment with MEK inhibitors, like trametinib (GSK1120212) (NCT00687622, Table 2). The most common side effects after those treatments include blood components' abnormalities, nausea, diarrhea, pyrexia, anorexia and fatigue. In the case of vemurafenib and dabrafenib treatment, some cases of squamous and basal cell carcinoma were recorded. The combinatorial treatment using a BRAF followed by a MEK inhibitor in melanoma patients, though lacking a significant difference in progression free or overall survival compared to monotherapy, came with a partial or, in some cases, even complete response of the patients (NCT01584648, Table 2). The side effects of these treatments were even milder than those in the monotherapy studied. Surprisingly, a reverse treatment study, using a MEK followed by a specific BRAF inhibitor, demonstrated some encouraging results by reporting an increase in TTP (time to progression) in melanoma patients [154].

However, the BRAFV600E mutation clearly differs from wtBRAF in that V600E is independent of RAS signalling and elevates basal kinase activity without KRAS mutations, while wtBRAF is RAS-dependent and are not mutually exclusive with KRAS mutations. As the V600E mutation accounts for 80% of all BRAF mutations, it explains the focus of search on the V600E-specific molecular inhibitors [27, 155].

### Combination of BRAF and MEK inhibitors

Because acquired resistance to BRAF inhibitors can lead to sustained mitogen-activated protein/extracellular signal–regulated kinase (MEK) activation in the presence of compound, therefore the combination of BRAF and MEK inhibitors may enhance growth inhibition. This combination may also deter the outgrowth of resistant melanoma cells by inhibiting the pathway at two different levels. As such, the combination of MEK 1/2 inhibitor GSK1120212 (GSK212) and BRAF inhibitor GSK2118436 (GSK436) was first tested in the phase I/II trials. The combination proved safe as no squamous cell carcinoma and decreased frequency of rash appeared, but only the randomized phase II section yielded a high response rate [148]. Furthermore, two randomized, phase 3 trials, have been initiated involving patients with metastatic melanoma and Trametinib and Dabrafenib Combination Therapy (ClinicalTrials.gov numbers, NCT01584648 and NCT01597908). There is also a three-part Phase 1/2 study to investigate the safety, pharmacokinetics, pharmacodynamics and clinical activity of trametinib (GSK1120212) and dabrafenib (GSK2118436) when administered in combination with the anti-EGFR antibody panitumumab in subjects with BRAF V600E or V600K positive CRC (ClinicalTrials.gov number, NCT01750918).

The combination of vemurafenib and the MEK inhibitor cobimetinib (GDC-0973) has been tested in a phase I trial with BRAF positive metastatic melanomas. The combination prooved tolerable and adverse events were manageable. Early data in eight vemurafenib-naïve patients showed tumour reduction but safety rather than efficacy was the purpose of this study and further research on efficacy is warranted. Two dose levels were selected for phase III investigation [149].

Initial results from a phase I/II trial of the combination of LGX818 and the MEK inhibitor MEK162 [150] in patients with BRAFV600-mutant metastatic melanoma were recently reported. This trial specifically allowed any BRAFV600 mutation. Initial results are encouraging with a hight response rate, no pyrexia, photosensitivity, or SCC have been reported to date. A phase III trial of combined LGX818 and MEK162 versus vemurafenib is planned.

In summary pharmacological attempts to inhibit a mutant GTP-ase like KRAS were hardly successful and only recently have provided new promise, rendering inhibitors of the key downstream effectors of RAS and targets the only alternative. Thereby, selective BRAFV600E and MEK inhibitors were developed.

### PI3K/PTEN/mTOR and RAS pathway

Apart from the Ras/Raf/MEK/ERK/MNK pathway, effective inhibitors specific for many of the key components of the Ras/PI3K/PTEN/mTOR have also been developed since both of these pathways are often implicated in therapeutic resistance and interact with other pathways [39].

Some colorectal tumours have both KRAS and PI3K pathway mutations. Two major differences between these KRAS and PI3K pathways have been observed. First, KRAS can activate also other substrates and not only PI3K/AKT/mTOR pathway, like BRAF/MEK/ERK pathway, Rho and Ral pathways. Moreover, PI3K/AKT/mTOR can be also regulated by other growth factor RAS independent signals. RAS and PIK3CA, the regulatory unit of PI3K, depending on the tissue and tumour, may or may not be considered components of the same pathway, although they can cross-talk in many cases. In general, if both KRAS and PIK3CA are mutated, this can provide an additive activation of the PI3K/AKT/mTOR signaling pathway. The presence of both mutations should be considered for predicting therapeutic response to targeted MEK of PI3K cancer therapeutics.

The p21-activated protein kinase 1 (PAK1) also interacts with the Ras/Raf/MEK/ERK and the PI3K/PTEN/Akt/mTOR cascades. PAK signaling molecules are downstream effectors of Rho family GTPase and interact with both Raf and Akt. Interestingly, IPA3 specific PAK inhibitor is able to inhibit the proliferation of melanoma and CRC cells with mutations at KRAS or NRAS better, than those containing mutations at BRAF. In contrast, treatment of cells with IPA3 or ectopic expression of DN PAK1 was required to sensitize RAS mutated cells to the BRAF inhibitor GDC-0897 or the MEK inhibitor ZD6244 [156].

### Target-specific cancer therapeutics: an assessment for KRAS and BRAF mutations

KRAS and BRAF mutations lead to the constitutive activation of EGFR signalling through the oncogenic RAS/RAF/MEK/ERK pathway. In a previous study using MEK (a downstream effector of KRAS and BRAF) inhibitors, it was demonstrated that BRAF mutant cell lines responded differently than those bearing mutant KRAS, raising the possibility that KRAS and BRAF mutant cancer cells might be differentially dependent on signalling mechanisms that involve MEK, a scenario also supported by the mutual exclusivity of these two mutations [157]. Treatment of patients with metastatic colorectal cancer (mCRC) has remarkably improved the onset of target-specific cancer therapeutics. For starters, three monoclonal antibodies have been approved, including cetuximab (Erbitux, ImClone) and panitumumab (Vectibix, Amgen), which are monoclonal antibodies against EGFR and bevacizumab (Avastin, Genentech), which is a monoclonal antibody against vascular endothelial growth receptor VEGFR. However, mutations in KRAS and BRAF genes have been associated with primary resistance to anti-EGFR monoclonal antibodies in CRC and targeted EGFR therapy [158].

**KRAS:** Currently, KRAS is the only potential biomarker for predicting the efficacy of anti-EGFR therapies in CRC. Recent trials have shown that patients with KRAS mutant tumours do not respond to anti-EGFR agents (cetuximab/panitumumab) because the activating mutation occurs downstream from the target of anti-EGFR therapy [51, 159]. It has been suggested that the resistance mutations in KRAS and other genes were highly likely to be present in a clonal subpopulation within the tumours prior to the initiation of panitumumab therapy [159–162].

**EGFR and KRAS:** Approximately 30% to 50% of colorectal tumours are known to have a mutated (abnormal) KRAS, indicating that up to 50% of patients with CRC might respond to anti-EGFR antibody therapy, they only have a reasonable opportunity to derive clinical benefit from the therapy. In addition, about 40% to 60% of patients with wild-type KRAS tumours do not respond [163]. It is highly recommended that all patients with mCRC who are candidates for anti-EGFR monoclonal antibody therapy have their tumours tested for KRAS mutation [77, 88, 164–167].

Nonetheless, a recent report suggested that the use of cetuximab or bevacizumab as a first-line therapy was associated with survival benefit among patients with p.G13D-mutated tumours, whereas KRAS codon 12 mutations were associated with resistance to cetuximab among chemotherapy-refractory CRC patients [81, 82, 169]. On the other hand, even with KRAS mutational testing, there are still many patients with KRAS wild-type tumours that do not respond to treatment with cetuximab or panitumumab [77, 88, 165, 169]. This suggests that other factors such as alterations in other EGFR effectors, including members of the RAS-mitogen activated protein kinase (MAPK) or phosphoinositide 3-kinase (P13K) pathways, could drive resistance to anti-EGFR therapy [30]. Moreover, previous reports have shown that RAS mutations are not found in lung cancers from patients with acquired resistance to EGFR TKIs [150, 173]. On the other hand, mutations in NRAS, a RAS family member and MEK1 have been shown to mediate acquired resistance to the mutant BRAF kinase inhibitor, vemurafenib, in melanoma cell lines [171, 172].

**BRAF:** Although the presence of mutated BRAF, which is present in 5% to 10% of colon tumours, highlights the potential adverse prognostic factors for stage II and III disease and patients across all disease stages [90, 174], patients with BRAF-mutated tumours do not experience a survival benefit from treatment with anti-EGFR mAb. None of the responders have BRAF mutations and show prolonged progression-free and OS compared with BRAF mutant patients [175, 204] Table 1.

**EGFR and BRAF:** Mutations in BRAFV600E predict a lack of response in wild-type KRAS mCRC treated with anti-EGFR MoAbs as well as poor prognosis. It is suggested that BRAF mutation may be used as an additional biomarker for the selection of mCRC patients who might benefit from anti-EGFR MoAbs therapy [30, 128, 175, 176]. Similarly, a retrospective pooled analysis of the two pivotal cetuximab studies demonstrated that the best treatment outcomes were observed in patients with both KRAS wild-type and BRAF wild-type tumours, but there were too few patients with BRAF mutations to determine whether BRAF mutation status alone could predict response to therapy [167]. Although results from these and other retrospective studies are compelling, there are as yet no prospective data that can help define the role of BRAF status in response to EGFR inhibitor therapy. Combined mutational analysis of both KRAS and BRAF could be used to prospectively select mCRC patients eligible for EGRF-targted monoclonal antibody treatment, Table 2. Likewise, BRAF mutations have also been found in patients with acquired resistance to imatinib in KIT/PDGFRα-mutant gastrointestinal stromal tumours (GISTs) [177].

In summary, the possibility that in KRAS and BRAF mutant cancer cells differential signalling mechanisms that involve MEK supported the mutual exclusivity of these two mutations, lead to the target-specific cancer therapeutics in the form of monoclonal antibodies against EGF and VEGF receptors. So far KRAS is the only potential biomarker for predicting the efficacy of anti-EGFR therapies in CRC, since KRAS mutant tumours do not respond to anti-EGFR agents. Presence or absence of mutant BRAF was not correlated to any survival benefit from treatment with anti-EGFR mAb.

Taking the above under consideration, patients with specific BRAF and KRAS mutations who failed initial treatment, should be further screened for other mutations within the same pathways, both in the same or in additional genes like PI3KCA, MEK and EGFR. According to the test results and taking also in account the disease stage and other clinical parameters, different approaches could be performed, including combinatorial drug treatments or combinations between immunotherapy or chemotherapy and/or drug treatment.

### Vemurafenib (PLX4032) and BRAF oncogene

PLX4032 and its analogs like PLX4720 have demonstrated selectivity between highly homologous wild-type BRAF and RAF1, and some selectivity for BRAFV600E compared to non-mutated BRAF. These compounds inhibit the inactive form of the MAP kinase domain by firmly anchoring in the ATP-binding site of BRAFV600E mutant activated cells Subsequent effects on proliferation and apoptosis are also entirely restricted to cells harboring BRAF mutations [178]. Nevertheless, BRAF mutations select patients with advanced melanoma for treatment with anti-BRAF agents (vemurafenib-PLX4032 and dabrafenib-GSK2118436). Addition of a phenyl ring for pharmacokinetic reasons to PLX4720, gave rise to vemurafenib, an FDA approved drug against BRAFV600E melanomas [179]. The collective data strongly suggest that PLX4720 initiates an apoptotic response only in cells with the V600E mutation. The same inhibitor unexpectedly activated the same MAPK pathway in KRAS mutant cells. In these cells, PLX4720, increase pMEK and pERK levels and therefore should be avoided in cancers caused by RAS mutants, alternatively, MEK inhibitors may be a more appropriate choice of therapeutic agent. Regardless of the agent chosen, it will probably need to be used in combination with another drug to effectively treat RAS-mutant tumours [180]. In melanoma models, PLX4720 induces cell cycle arrest and apoptosis exclusively in BRAFV600E-positive cells. It should be noted that in melanoma, BRAF and NRAS mutants can in some cases coexist in the same tumour in contrast with colorectal cancer [44, 181, 182]. A high proportion of patients with metastatic melanoma have shown clinical responses when treated with an inhibitor against the BRAFV600E [183], (Table 2).

**Figure 6:**
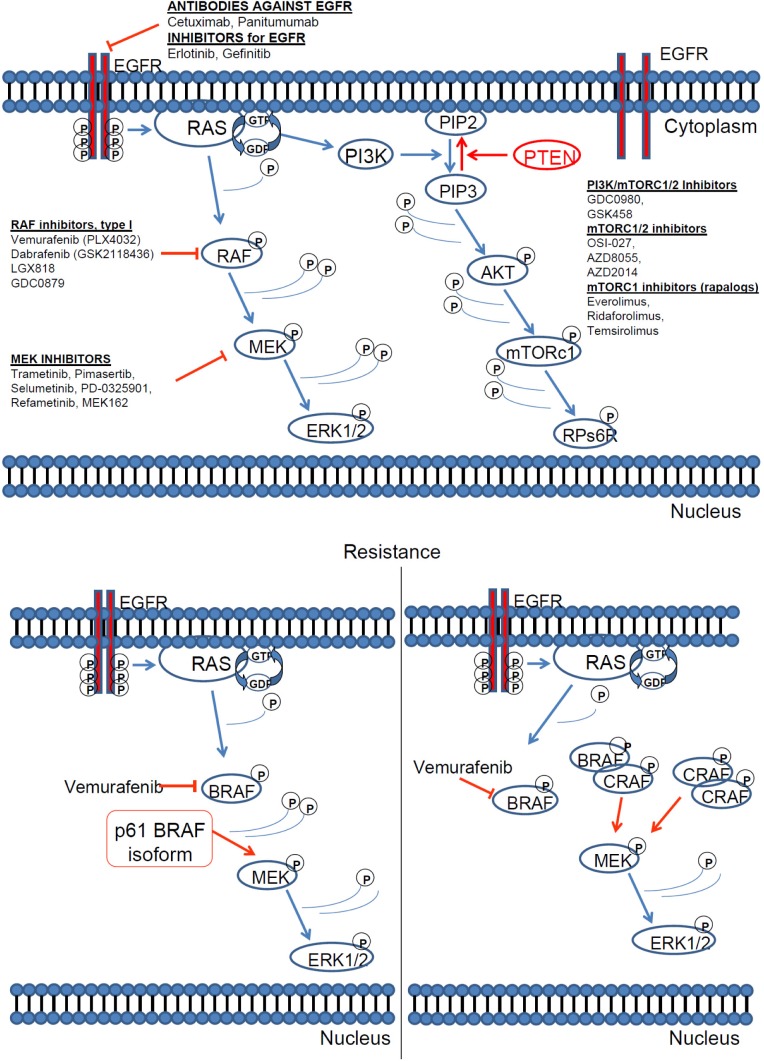
RAS pathway inhibitors and resistance mechanisms [160, 161, 180]. (**A**) Inhibitors of the key downstream effectors of RAS. All downstream RAS effectors are cytosolic protein kinases that form a tiered protein kinase cascade downstream of RAS. (**B**) The resistance mechanism against BRAF inhibitor vemurafenib (PLX4032).

Nevertheless, soon after the success story of PLX4032, a new resistance mechanism against BRAF inhibitor vemurafenib (PLX4032) was discovered. Cells become resistant to vemurafenib (PLX4032) with different mechanisms, most notably they express a 61-kDa variant form of BRAFV600E, p61BRAFV600E, which lacks exons 4–8, a region that encompasses the RAS-binding domain. In cells in which p61BRAFV600E is expressed endogenously or ectopically, ERK signalling is resistant to the RAF inhibitor [184, 209], (Figure 6). Other resistance mechanisms include upregulation of RTK or NRAS [171]. It is of interest that BRAFV600E mutant colorectal cancers are non responsive to PLX4032 due to, among other reasons, a feedback activation of EGFR [185]. Parallel inhibition of other oncogenic pathways like PI3K, can sensitise resistant BRAFV600E colorectal tumour cells to PLX4720 treatment [186].

### The impact of KRAS vs BRAF oncogenes on their differential response to other BRAF and MEK inhibitors

**GSK2118436 inhibitor and BRAF:** Other mutant BRAF inhibitors are also gaining momentum clinically, such as GSK2118436 (Dabrafenib), and are closely monitored in the melanoma field. GSK2118436 is another ATP competitive, reversible inhibitor of mutant BRAFV600E, as well as V600D/K and V600G kinases. GSK2118436 is a potent BRAF inhibitor with high selectivity for mutant BRAF compared with the wild-type protein. This drug inhibits intratumoural phosphorylated ERK levels, indicating MAPK pathway suppression. Interestingly, the BRAF and MEK inhibitor combination (GSK2118436 and GSK1120212) demonstrated a potential reduction in drug resistance in melanoma [187], (Table 2).

### MEK inhibitor CI-1040 and BRAF

The most frequent genetic abnormalities in ovarian carcinoma are mutations in KRAS, BRAF, and p53. Mutational status was correlated with growth inhibition and apoptosis induction by the MEK inhibitor CI-1040 that prevented activation of the downstream target, ERK1/2. CI-1040 significantly reduced cellular proliferation and induced apoptosis in cell lines with either KRAS or BRAF mutations in comparison with cell lines with wild-type sequences. The RAS–RAF–MEK–ERK pathway may play an important role in ovarian carcinogenesis but not in endometrial carcinogenesis. Similarly, alternative pathways for ERK activation, such as crosstalk with the PI3K pathway, exist in endometrial cancer but are rare in ovarian cancer [188] (Table 2).

Thus, the prognostic or predictive relevance of the KRAS and BRAF genotype in CRC remains controversial despite several investigations. The significance of KRAS/BRAF mutations as predictive or prognostic biomarkers should be taken into consideration when selecting a KRAS/BRAF screening assay. The availability of companion biomarkers should improve drug efficacy, decrease toxicity, and lead to a more individualized approach to cancer treatment.

### Oncogene induced senescence or organismal aging

It is common knowledge that cancer is an age-related disease. Overexpression of oncogenic RAS (HRAS or KRAS V12) or its downstream effector RAF can lead to senescent phenotypes called oncogene-induced senescence (OIS) [189, 190]. Cellular senescence is strongly correlated to organismal aging with the main difference between cancer and aging to be that the control of cell cycle is disabled in cancer [191]. Numerous agents targeting mTOR, PI3K, growth factor receptors, and related tyrosine kinases, RAS, RAF, and B-RAF, S6K, MEK1/2 have been tested to treat cancer. While RAF inhibitors are effective against melanomas with BRAFV600E mutations, they may induce keratoacanthomas and cutaneous squamous cell carcinomas by selecting for RAS-mutated cells. Intriguingly, only metformin, which affects the mTOR pathway, has been reported to not only reduce not only the risk of cancer, but of other age-related diseases as well [192, 193].

Previously, the mTOR was the only pathway known to be involved in acquiring classic markers of a senescent phenotype, including cyclin D1 accumulation. Recent studies have revealed an additional MEK/ERK pathway that is required for the acquisition of at least one hallmark of senescence: hyper-accumulation of cyclin D1 [194].

## DETECTION OF KRAS AND BRAF MUTATIONS BY DNA SEQUENCING

Screening and identification of KRAS mutation status is determined via polymerase chain reaction (PCR) amplification followed by dideoxy DNA sequencing or/and Pyrosequencing of formalin-fixed, paraffin-embedded block, unstained slides, or fresh snap frozen biopsy tissue for the presence of a mutation in codons 12, 13, 61 or 146 of the KRAS gene on chromosome 12. Likewise, BRAFV600E mutation status is also determined via PCR analysis of formalin-fixed, paraffin-embedded tissue. Dideoxy DNA sequencing is the most commonly used sequencing method; however, it may not detect a minority of mutant sequences present in a background of abundant wild-type DNA sequence [174]. Digital PCR described by Vogelstein and Kinzler [195], does offer the capability of quantifying mutant allele very accurately. However, its application to a large-scale study is currently limited because of the cost and labor intensive nature of this technology. Alternatively, the array-based KRAS mutation detection system described by Prix et al. [196], uses peptide nucleic acid-mediated PCR clamping followed by biochip array hybridization. Nonetheless, this method may fail to detect some rare mutations. Moreover, the applicability of this technique to paraffin-embedded tissue and its cost effectiveness and limited throughput remain important issues. Compared with dideoxy sequencing, Pyrosequencing assay offers simplicity and cost effectiveness, particularly in the setting of large-scale projects and clinical assays [197]. Pyrosequencing has been also reported as a very sensitive method able to detect genetic heterogeneity in tumours [198]. As an alternative to Pyrosequencing, laser capture microdissection technique can collect pure population of tumour cells to increase sensitivity for DNA sequencing. However, performing laser capture micro dissection is time consuming and labor intensive and yields less of DNA than manual tissue dissection, thereby limiting the amount of biomarkers that can be investigated.

### Clinical testing for KRAS and BRAF mutations

Other clinical applications also require highly sensitive mutation detection, for instance, the monitoring of minimal residual disease after treatment, monitoring of relapse caused by the emergence of resistance mutations, and identification of somatic mutations in early tumourigenesis [199]. For these reasons, the development of sensitive, reliable, and cost-effective methods for mutation testing is of paramount importance. Genotyping for ‘driver mutations’ is becoming increasingly central to oncology care and currently in United States performed using a multiplexed PCR-based SNaPshot assay plus FISH for ALK translocations for NSCLC patients as part of standard care named Competitive Amplification of Differentially Melting Amplicons (CADMA) [200]. While widely agreed that it is important to identify patients with EGFR and ALK given the availability of effective therapeutics, it is also noteworthy that in a short time frame a large amount of patients will be diagnosed for less common mutations like BRAF, PIK3CA and HER2, which are also have relevant candidate targeted therapies [201].

## CONCLUSIONS AND FUTURE DIRECTIONS

Frequently reported evidence from experimental and clinical studies has demonstrated that signalling by KRAS and BRAF oncogenes can present similarities but also differential oncogene effects. Therefore differential KRAS versus BRAF pathways, their components and biological effects have attracted much attention as promising targets and markers for targeted therapeutics. In detail:

KRAS, a more frequent mutation than BRAF in CRC is mostly distributed along the proximal-distal axis of the colorectum, has little prognostic value as compared to BRAF in overall patient survival and a mutation more likely to develop on the right of the colon. BRAF is highly associated to metastasis, MSI events and appears to be a strong prognostic factor for overall patient survival.

BRAF mutations seem to occur more frequently in CRC tumours with MSI-high characterised by dMMR, and poses as strong OS prognostic factor, however presence of KRAS mutations may suggest highest recurrence rate.

Pharmacological attempts to inhibit a mutant GTP-ase like KRAS were hardly successful and only recently have provided new promise, rendering inhibitors of the key downstream effectors of RAS and targets the only alternative. Thereby, selective BRAFV600E and MEK inhibitors were developed.

The possibility that in KRAS and BRAF mutant cancer cells differential signalling mechanisms that involve MEK, supported the mutual exclusivity of these two mutations, lead to the target-specific cancer therapeutics in the form of monoclonal antibodies against EGFR and VEGF receptors. So far KRAS is the only potential biomarker for predicting the efficacy of anti-EGFR therapies in CRC, since KRAS mutant tumours do not respond to anti-EGFR agents. Presence or absence of mutant BRAF was not correlated to any survival benefit from treatment with anti-EGFR mAb.

The prognostic or predictive relevance of the KRAS and BRAF genotype in CRC remains controversial despite several investigations. The significance of KRAS/BRAF mutations as predictive or prognostic biomarkers should be taken into consideration when selecting a KRAS/BRAF screening assay. The availability of companion biomarkers should improve drug efficacy, decrease toxicity, and lead to a more individualized approach to cancer treatment.

However, many questions remain to be answered: such as the tumour specific per oncogene effects, the role of each oncogene in tumour heterogeneity, as well as resistance mechanisms to drugs targeting either oncogene or components of the so-called RAS pathway.

Furthermore cross-talk between RAS and BRAF oncogenes with other mutated pathways like PI3K and APC will provide new molecular mechanisms and will assist the design of efficient rational combinatorial anti-cancer protocols for personalised therapy.
